# Comparison in trends and outcomes of multiple vs. single arterial coronary bypass graft surgery

**DOI:** 10.3389/fcvm.2025.1661006

**Published:** 2025-11-05

**Authors:** Qiuju Ding, Han Li, Xiaofeng Cheng, Min Ge, Qing Zhou

**Affiliations:** ^1^ Department of Cardio-Thoracic Surgery, Nanjing Drum Tower Hospital, The Affiliated Hospital of Nanjing University Medical School, Nanjing, China; ^2^ Department of Cardio-Thoracic Surgery, Nanjing Drum Tower Hospital, Clinical College of Nanjing University of Chinese Medicine, Nanjing, China

**Keywords:** coronary artery bypass grafting (CABG), single arterial grafting (SAG), multiple arterial grafting (MAG), in-hospital mortality, short-term

## Abstract

**Objective:**

Multiple arterial grafting (MAG) has been suggested to confer long-term survival benefits for patients undergoing coronary artery bypass grafting (CABG), yet its short-term benefits remain uncertain. This study aims to analyze the impact of MAG on in-hospital outcomes and identify potential risk factors.

**Methods:**

A retrospective analysis was conducted from all patients who underwent CABG surgery in our development from January 2022 to December 2024. A generalized mixed-effects model and sensitivity analysis were employed to evaluate the influence of the type of CABG bypass graft on in-hospital major adverse cardiac and cerebrovascular events (MACCEs), postoperative dialysis, intra-aortic balloon pump (IABP) use, re-thoracotomy for bleeding and sternal wound infection (SWI).

**Results:**

A total of 960 patients were included in this study. Patients who received MAG surgeries had more coronary artery lesions observed preoperatively. Compared with patients who underwent single arterial grafting (SAG), those who received MAG surgery did not show significant differences in the incidence of in-hospital MACCEs, postoperative dialysis, IABP use, re-thoracotomy or SWI. Interestingly, good left ventricular function was associated with a reduced occurrence of postoperative dialysis, MACCEs, and IABP application. Chronic renal insufficiency emerged as a risk predictor of major in-hospital adverse events.

**Conclusion:**

This single-center study did not find significant differences in short-term outcomes between MAG and SAG groups. However, caution should be exercised when applying these findings to other clinical environments and patient populations. Further multi-center, prospective randomized controlled trials (RCTs) are needed to validate and extend our results.

## Introduction

Coronary artery bypass grafting (CABG) remains a cornerstone surgical approach for treating coronary artery disease (1). In recent years, with in-depth research into the long-term patency of grafts and their clinical benefits, multiple arterial grafting (MAG) strategies, such as the use of bilateral internal mammary arteries (BIMA) and radial arteries (RA), have emerged as a research focus in the field of coronary revascularization due to their potential long-term advantages (2, 3). Compared with traditional single arterial grafting, such as left internal mammary artery (LIMA) combined with saphenous vein graft (SVG), MAG is believed to offer superior graft patency (4, 5), thereby significantly reducing the risk of long-term cardiovascular events and improving patient survival rates (6–8).

Although several observational studies and meta-analyses have suggested that MAG strategies may confer substantial long-term benefits (6, 7, 9, 10), the Society of Thoracic Surgeons (STS) (11) database indicate that the adoption rate of MAG remains below 5%, partly attributable to concerns among surgeons regarding perioperative risks. The surgical complexity of MAG strategies, such as prolonged cardiopulmonary bypass and aortic cross-clamp times, may increase in-hospital risks. Le et al. reported that MAG was associated with significantly elevated postoperative troponin T levels, suggesting a potential higher risk of myocardial injury, although no significant differences were observed in in-hospital mortality, stroke, or sternal wound infection (SWI) rates compared with the SAG group (12). Nevertheless, the limited sample size (*n* = 58) in that study precluded a comprehensive evaluation of the impact of MAG on in-hospital outcomes. Furthermore, variability in surgeon preferences, patient comorbidities (e.g., diabetes mellitus, renal insufficiency), and graft selection may confound comparisons of in-hospital outcomes between MAG and SAG strategies (13).

In this study, we stratified by the type of bypass grafts performed, systematically compared the in-hospital outcomes of MAG and SAG strategies on CABG surgery, including major adverse cardiac and cerebrovascular events (MACCEs), postoperative dialysis, the use of intra-aortic balloon pump (IABP), re-thoracotomy for bleeding and SWI, rigorously assessing their safety and early effectiveness. We seek to provide higher-level evidence to guide the selection of grafting strategies in clinical practice while exploring potential factors influencing in-hospital outcomes to optimize surgical decision-making in CABG.

## Methods

### Population and study design

This retrospective cohort study was conducted at Department of Cardio-thoracic Surgery, Nanjing Drum Tower hospital. The Ethics Committee of Nanjing Drum Tower hospital approved this study and all of the subjects gave written informed consent.

Patients suffering from severe multivessel coronary artery disease involving the left coronary arteries (with stenosis >75%) or with left main coronary artery obstruction exceeding 50% received CABG. According to the 2021 ACC/AHA/SCAI guidelines (1), the LIMA is prioritized as the preferred graft for the left anterior descending artery (LAD) due to its superior long-term patency and significant mortality reduction. For younger, low-risk patients, the right internal mammary artery (RIMA) or RA is preferred over the SVG, given their better long-term patency rates. SVG remains suitable for complex or multivessel coronary artery disease, though its long-term patency is lower, necessitating strict postoperative management with antiplatelet therapy and lipid-lowering treatment to delay atherosclerotic progression. All surgeries were operated electively by experienced surgeons in our department. The decision of graft type selection is systematically guided by a multidisciplinary cardiac team through comprehensive case discussions. All decisions are made with explicit patient informed consent, ensuring transparency and respect for individual treatment preferences.

All patients undergoing isolated CABG surgeries from 2022 to 2024 in our department were eligible to enter the study. Those with any of the following conditions were excluded: Patients with sequential LIMA grafts were excluded to avoid ambiguity in defining a multi-arterial bypass graft, as sequential LIMA grafts involve one arterial conduit anastomosed to multiple stenotic lesions (i.e., it would be challenging to categorize if these patients have received one arterial graft or two arterial grafts). Patients who received only venous grafts were also excluded, as they did not fall into either the SAG or MAG (14) categories. Additionally, patients with any previous cardiac surgery (CABG or others) were excluded due to the potential for different outcomes compared to those undergoing their first CABG. Previous surgeries can cause scar tissue and adhesions, relocations of blood vessels, and changes in heart chambers' anatomy, all of which can complicate subsequent operations and impact outcomes, and they may also indicate more severe underlying cardiovascular disease. Finally, we excluded patients with severe comorbidities that would significantly increase the surgical risk, such as end-stage liver or kidney disease, typically associated with poorer prognoses. Accordingly, 960 patients comprised the final study cohort: SAG (*n* = 703; 73.2%) and MAG (*n* = 257; 26.8%).

### Variable definition and end points

The preoperative, intraoperative, and postoperative clinical data were collected in both groups. The preoperative clinical data were age, gender, body mass index (BMI), histories of hypertension, chronic kidney disease (CKD), diabetes, chronic obstructive pulmonary disease (COPD), pulmonary disease, hypertension, valvular disease, atrial fibrillation, peripheral vascular disease, smoking, drinking, recent myocardial infarction (MI), prior percutaneous coronary intervention (PCI), grade of cardiac function and the condition of vascular lesions. Intraoperative indicators include: type of bridging vessels, and whether they are under extracorporeal circulation. Postoperative clinical data were mechanical ventilation duration, length of stay in the CICU. Endpoint: in-hospital mortality, stroke, MACCEs, SWI, IABP application, postoperative dialysis, re-thoracotomy for bleeding, and the total length of hospital.

### Statistical analysis

Categorical data are presented as frequencies and compared using a Chi-squared test. Non-normally distributed data were averaged as a median with an IQR and analyzed using the Wilcoxon rank sum test. The Bonferroni test was used to correct for multiple corrections between the groups.

To estimate the associations between type of graft (reference group: SAG) and outcomes, namely mortality, MACCEs, IABP use, stroke, re-thoracotomy for bleeding and the requirement for postoperative dialysis, we conducted a generalized mixed-effects multivariable logistic regression, using the lme4: Linear Mixed-Effects Models using “Eigen” and S4 R package (15), with no data imputation. The model we selected represents an extension of logistic regression models, incorporating both fixed and random effects. The confounding factors included in the model as fixed effects were age, sex, neurological dysfunction, renal dysfunction, recent MI, pulmonary disease, New York Heart Association (NYHA) class IV, pulmonary hypertension, diabetes, left main stem disease, the degree of left ventricular dysfunction, peripheral vascular disease, and MAG. Random effect for patient was specified to address clustering. Sensitivity analysis was conducted by sequentially excluding each covariate from the fixed-effects model to assess the robustness of the estimated effects. The statistical analysis was performed using R software (Version 4.4.0), with *P*-value of <0.05 was considered statistically significant.

## Results

We analyzed data from a total of 960 patients and divided them into two groups respectively, MAG and SAG, based on the type of bypass graft. There were 257 patients who received MAG surgery and 703 patients who underwent SAG surgery. Nearly 21.9% of the surgeries in this study were performed on-pump, with the rate balanced between MAG and SAG cohorts (23.7% vs. 21.2%, *p* = 0.45, respectively). Compared with the SAG group, the MAG group had a higher proportion of male patients (80.9% vs. 74.1%, *p* = 0.035), a younger median age (median age of 59 vs. 68, *p* < 0.001), and a higher median BMI (median BMI of 24.98 vs. 24.22, *p* = 0.004). The proportions of patients with a history of smoking and alcohol consumption were similar in both groups, and the proportions of patients with NYHA class IV heart function and severe LVEF dysfunction were balanced between the two groups.

Regarding comorbidities, the MAG group had a lower proportion of patients with a history of stroke (12.1% vs. 21.3%, *p* = 0.002) and a lower prevalence of hypertension (64.6% vs. 72.3%, *p* = 0.026), but a higher prevalence of hyperlipidemia (37.4% vs. 26.6%, *p* = 0.002). The MAG group also had a higher proportion of patients with a recent history of MI (26.5% vs. 19.8%, *p* = 0.032), while the proportion of patients with a prior PCI was similar between the two groups. The prevalence rates of chronic renal insufficiency, diabetes mellitus, valvular heart disease, atrial fibrillation, peripheral arterial disease, COPD, and interstitial pneumonia showed no significant differences between the two groups. The proportions of patients with left main and left anterior descending artery lesions were similar between the two groups, but the MAG group had a higher proportion of patients with three vessel disease (79.4% vs. 69.4%, *p* = 0.003), including those involving the left circumflex artery (90.3% vs. 78.5%, *p* < 0.001) and the right coronary artery (88.7% vs. 81.1%, *p* = 0.007). The EuroScore II score was lower in the MAG group compared with the SAG group (median 3% vs. 4%, *p* = 0.003).

Compared with patients in the SAG group, patients in the MAG group had shorter durations of mechanical ventilation (median 6.5 h vs. 8.0 h, *p* < 0.001) and shorter stays in the cardiac intensive care unit (CICU) (median 2d vs. 3d, *p* = 0.036).

The overall in-hospital mortality rate was 0.7%, but there was no significant difference in in-hospital mortality between patients who underwent MAG surgery and those in the SAG group. The overall incidence of MACCEs was 3.4%, with no significant difference between the two groups. Other secondary outcomes, including in-hospital SWI, IABP use, postoperative dialysis, and re-thoracotomy for bleeding, showed similar proportions between the two groups. Moreover, MAG did not significantly prolong the length of hospital stay (Table 1).

**Table 1:** Unadjusted outcomes stratified by types of grafts performed.

Outcome	Overall	MAG	SAG	*p*-value^b^
	*N* = 960^a^	*N* = 257^a^	*N* = 703^a^	
MACCE	33 (3.4%)	9 (3.5%)	24 (3.4%)	>0.999
Stroke	14 (1.5%)	4 (1.6%)	10 (1.4%)	>0.999
Mortality	7 (0.7%)	1 (0.4%)	6 (0.9%)	0.749
Sterile wound dehiscence	43 (4.5%)	12 (4.7%)	31 (4.4%)	>0.999
IABP application	21 (2.2%)	5 (1.9%)	16 (2.3%)	0.952
Re-thoracotomy	12 (1.3%)	2 (0.8%)	10 (1.4%)	0.64
Postoperative dialysis	29 (3.0%)	4 (1.6%)	25 (3.6%)	0.165
Total length of hospital stay (d)	20.0 (17.0, 25.0)	20.0 (16.0, 25.0)	21.0 (17.0, 25.0)	0.058

MACCE, major adverse cardiac and cerebrovascular events; IABP, intra-aortic balloon pump.

^a^ Continuous variables are presented as Median (Q1, Q3); Categorical variables as *n* (%). *P*-values were calculated using Wilcoxon rank-sum test for continuous variables and Chi-square test for categorical variables.

^b^ Pearson's Chi-squared test; Wilcoxon rank sum test.

**Figure 1:**
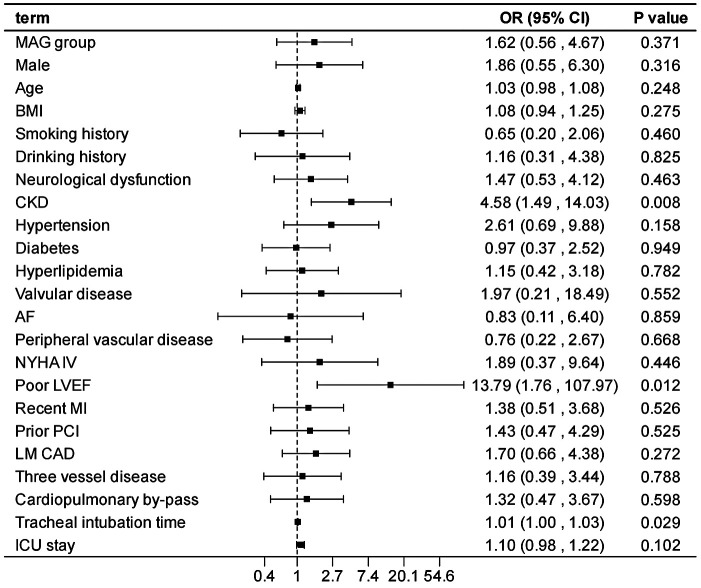
Effect estimates were calculated using a generalized mixed-effects model to establish the effect of specific perioperative covariates upon in-hospital MACCE in patients receiving CABG.

**Figure 2:**
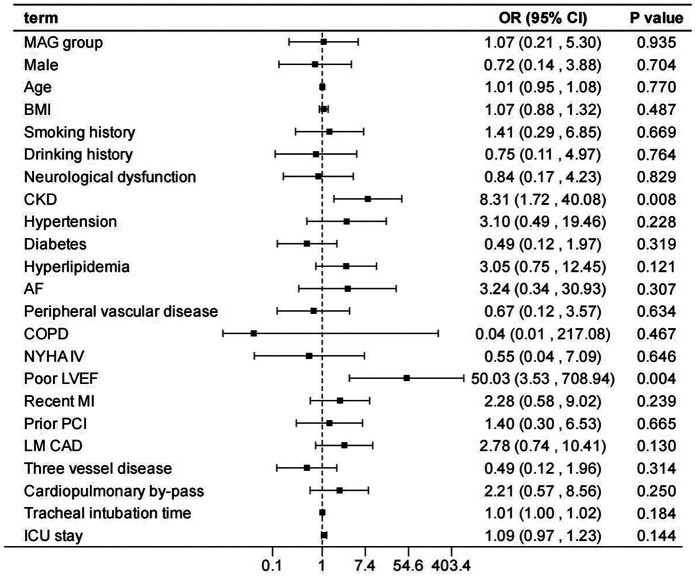
Effect estimates were calculated using a generalized mixed-effects model to establish the effect of specific perioperative covariates upon IABP use in patients receiving CABG.

**Figure 3:**
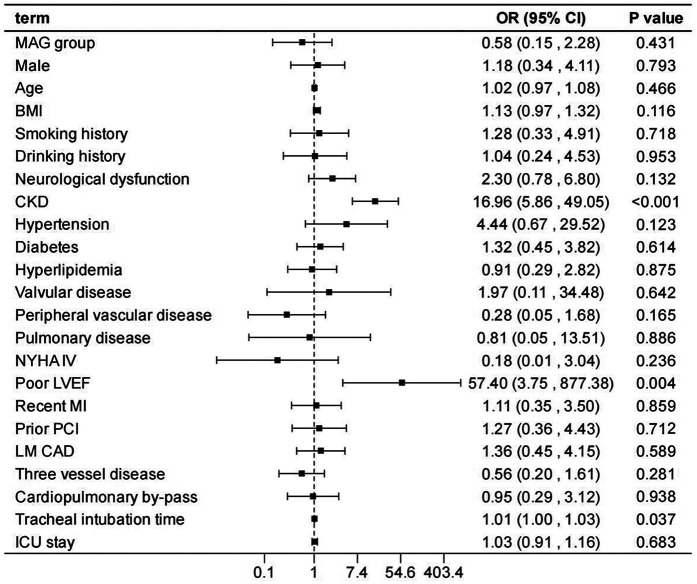
Effect estimates were calculated using a generalized mixed-effects model to establish the effect of specific perioperative covariates upon postoperative dialysis in patients receiving CABG.

**Figure 4:**
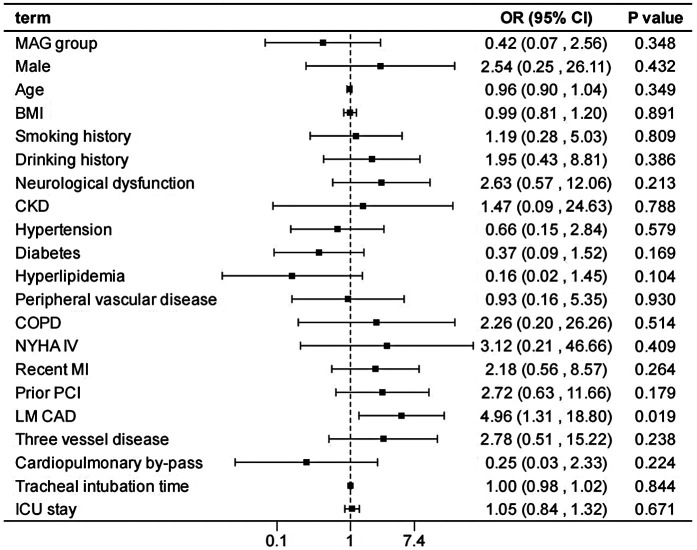
Effect estimates were calculated using a generalized mixed-effects model to establish the effect of specific perioperative covariates upon re-thoracotomy for bleeding in patients receiving CABG.

**Figure 5:**
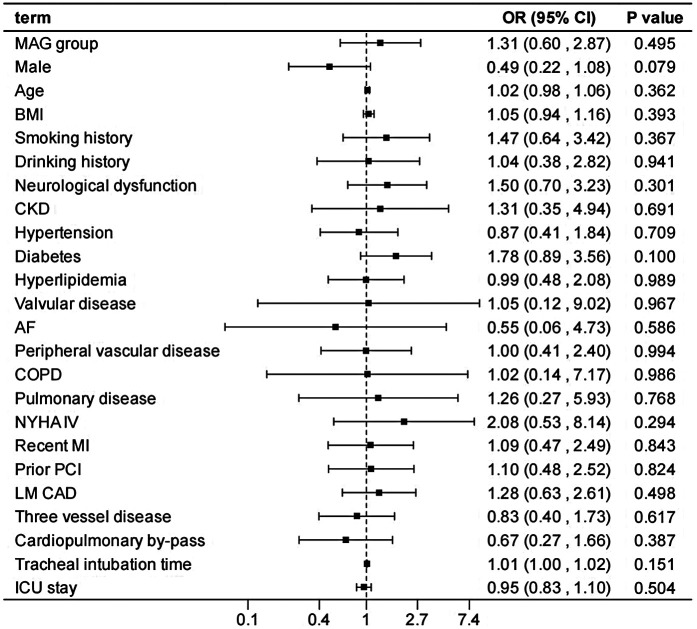
Effect estimates were calculated using a generalized mixed-effects model to establish the effect of specific perioperative covariates upon SWI in patients receiving CABG.

After adjusting the model for all the covariates detailed in Table 2, we found that, compared with SAG group, the patients receiving MAG surgery had no impact on post-operative MACCEs, postoperative dialysis, IABP use, re-thoracotomy for bleeding and SWI (*p* > 0.05; Figures 1–5; Supplementary Tables S1–S5). Interestingly, chronic renal insufficiency was closely associated with multiple adverse outcomes, including MACCEs (OR = 4.58, 95% CI: 1.49–14.03, *p* = 0.008; Figure 1; Supplementary Table S1), IABP application (OR = 8.31, 95% CI: 1.72–40.08, *p* = 0.008; Figure 2; Supplementary Table S2), and postoperative dialysis (OR = 16.96, 95% CI: 5.86–49.05, p < 0.001; Figure 3; Supplementary Table S3). On the other hand, a poor LVEF could, to a certain extent, increase the occurrence of MACCEs (OR = 13.79, 95% CI: 1.76–107.97, *p* = 0.012; Figure 1; Supplementary Table S1), IABP application (OR = 50.03, 95% CI: 3.53–708.94, *p* = 0.004; Figure 2; Supplementary Table S2), and postoperative dialysis (OR = 57.40, 95% CI: 3.75–877.38, *p* = 0.004; Figure 3; Supplementary Table S3). The duration of mechanical ventilation was correlated with MACCEs (OR = 1.01, 95% CI: 1.0–1.03, *p* = 0.029; Figure 1; Supplementary Table S1) and postoperative dialysis (OR = 1.01, 95% CI: 1.0–1.03, *p* = 0.037; Figure 3; Supplementary Table S3). Concomitant left main coronary artery disease served as a risk predictor for re-thoracotomy for bleeding (OR = 4.96, 95% CI: 1.31–18.8, *p* = 0.019; Figure 4; Supplementary Table S4).

**Table 2:** Baseline characteristics.

Variable	Overall	MAG	SAG	*p*-value^a^
	*N* = 960^a^	*N* = 257^a^	*N* = 703^a^	
Gender (male)	729 (75.9%)	208 (80.9%)	521 (74.1%)	**0**.**035**
Age (years)	66.00 (58.00, 71.00)	59.00 (54.00, 66.00)	68.00 (60.00, 73.00)	**<0**.**001**
BMI (kg/m^2^)	24.49 (22.49, 26.73)	24.98 (23.00, 26.99)	24.22 (22.31, 26.66)	**0**.**004**
Smoking history	353 (36.8%)	104 (40.5%)	249 (35.4%)	0.174
Drinking history	169 (17.6%)	54 (21.0%)	115 (16.4%)	0.114
Neurological dysfunction	181 (18.9%)	31 (12.1%)	150 (21.3%)	**0**.**002**
CKD (Cr > 133 mmol/L)	54 (5.6%)	9 (3.5%)	45 (6.4%)	0.117
Hypertension	674 (70.2%)	166 (64.6%)	508 (72.3%)	**0**.**026**
Diabtes	450 (46.9%)	123 (47.9%)	327 (46.5%)	0.767
Hyperlipidemia	283 (29.5%)	96 (37.4%)	187 (26.6%)	**0**.**002**
Valvular disease	24 (2.5%)	5 (1.9%)	19 (2.7%)	0.666
Atrial fibrillation	34 (3.5%)	5 (1.9%)	29 (4.1%)	0.155
Peripheral vascular disease	160 (16.7%)	38 (14.8%)	122 (17.4%)	0.397
COPD	34 (3.5%)	5 (1.9%)	29 (4.1%)	0.155
Pulmonary disease	40 (4.2%)	6 (2.3%)	34 (4.8%)	0.125
NYHA IV	41 (4.3%)	7 (2.7%)	34 (4.8%)	0.21
Good LVEF (EF > 50%)	698 (72.7%)	191 (74.3%)	507 (72.1%)	0.551
Moderate LVFF (EF 30%–50%)	252 (26.3%)	65 (25.3%)	187 (26.6%)	0.745
Poor LVFF (EF < 30%)	10 (1.0%)	1 (0.4%)	9 (1.3%)	0.398
Rencent MI	207 (21.6%)	68 (26.5%)	139 (19.8%)	**0**.**032**
Prior PCI	180 (18.8%)	46 (17.9%)	134 (19.1%)	0.753
Left main coronary artery	280 (29.2%)	74 (28.8%)	206 (29.3%)	0.941
Left anterior descending	941 (98.0%)	252 (98.1%)	689 (98.0%)	>0.999
Circumflex	784 (81.7%)	232 (90.3%)	552 (78.5%)	**<0**.**001**
Right coronary	798 (83.1%)	228 (88.7%)	570 (81.1%)	**0**.**007**
Three vessel disease	692 (72.1%)	204 (79.4%)	488 (69.4%)	**0**.**003**
Cardiopulmonary by-pass	210 (21.9%)	61 (23.7%)	149 (21.2%)	0.450
EuroScore II	0.04 (0.03, 0.04)	0.03 (0.02, 0.04)	0.04 (0.03, 0.04)	**0**.**003**
Tracheal intubation time (h)	7.50 (5.00, 15.50)	6.50 (4.50, 11.50)	8.00 (5.50, 16.00)	**<0**.**001**
Total length of stay in CICU (d)	2.50 (2.00, 4.00)	2.00 (2.00, 3.00)	3.00 (2.00, 4.00)	**0**.**036**

Values in bold indicate *p* < 0.05.

BMI, body mass index; CICU, cardiac surgical intensive care unit; CKD, Chronic kidney disease; COPD, chronic obstructive pulmonary disease; LVEF, left ventricular ejection fraction; IABP, intra-aortic balloon pump; MI, myocardial infarction; NYHA, New York Heart Association; PCI, percutaneous coronary intervention.

^a^ Continuous variables are presented as Median (Q1, Q3); Categorical variables as *n* (%). *P*-values were calculated using Wilcoxon rank-sum test for continuous variables and Chi-square test for categorical variables.

^b^ Pearson's Chi-squared test; Wilcoxon rank sum test.

In the sensitivity analysis by excluding each variable, the outcomes remain consistent with those of the main analysis (Supplementary Tables S6–S10), demonstrating the stability of the model.

## Discussion

This study conducted a detailed analysis of 960 patients undergoing CABG to comprehensively evaluate the differences in in-hospital outcomes between two distinct types of bypasses grafting procedures: MAG and SAG. Furthermore, it explored the impact of related factors on the incidence of postoperative adverse events. Our findings revealed that patients receiving MAG had more coronary artery lesions observed preoperatively; however, there was no significant difference observed in the risk of in-hospital adverse events between MAG and SAG groups. Additionally, chronic renal insufficiency, prolonged mechanical ventilation, and the presence of left main coronary artery disease were identified as potential risk factors for in-hospital adverse events, whereas a good left ventricular ejection fraction was beneficial in reducing the occurrence of such events.

Current research consistently demonstrates that patients undergoing MAG procedures benefit from long-term advantages, including superior graft patency (16), reduced long-term mortality (17, 18), and decreased cardiovascular mortality rates (19). However, data regarding short-term outcomes remain scarce. Study conducted by Damgaard et al., indicates that although MAG theoretically offers long-term benefits, there are no significant differences in mortality, rehospitalization rates, or major complication rates within the first postoperative year compared to SAG (20), suggesting that short-term outcomes may be more influenced by perioperative management and the patient's underlying health status. Muneretto et al. found no significant differences between MAG and SAG groups in in-hospital mortality, mechanical ventilation time, ICU stay, or hospital stay, but reported a lower incidence of early postoperative cerebrovascular accidents in the MAG group (21). Hwang et al. further pointed out that while MAG may theoretically offer better hemodynamic properties and lower restenosis rates, the increased surgical complexity can lead to higher intraoperative blood loss and prolonged operative times, potentially impacting short-term recovery (22). This is corroborated by findings from Le et al., who noted that in terms of in-hospital outcomes, MAG and SAG do not show a unified trend in key metrics such as mortality, stroke, MI, and repeat revascularization (12). Multiple large-scale studies have reported slightly higher in-hospital complication rates in the MAG group, possibly due to the technical challenges, patient selection, and intraoperative management (17, 23, 24). Overall, there is no consensus on in-hospital outcomes between MAG and SAG. Despite the long-term benefits of MAG, many surgeons prefer SAG strategy due to concerns over in-hospital adverse events, particularly in elderly patients, or those with multiple comorbidities, or those at higher surgical risk. After adjusting for all covariates, our findings showed no significant differences at short-term follow-up observed between MAG and SAG groups, except the influence from potential confounders, the possible explanations for this were as followed.

First, in the unadjusted two cohorts, an important observation was the difference between MAG and SAG cohorts in median age (59 vs. 68, *p* < 0.001) and EuroScore II (3% vs. 4%, *p* = 0.003). Despite adjustment for covariates, but the unadjusted data suggest that, MAG patients were younger and had lower surgical risk, which reduced the occurrence of complications to a certain extent, suggesting that short-term outcomes may be more influenced by the patient's underlying health status. Second, the first arterial graft (most commonly the LIMA) is anastomosed to the most important vessel (commonly the LAD) in our study. Occlusion of a graft in a coronary artery other than the LAD artery may not have a survival effect (25). Moreover, based on the preferences of the surgeons in our center, is that the radial artery was the preferred second arterial conduit rather than RIMA, whereas a high rate of use of radial artery grafts in the MAG group could have improved results in this group (26). Thus, during the initial postoperative period, the two surgical approaches have similar effects in improving patients' symptoms and facilitating recovery, although patients receiving MAG had more severe coronary artery disease preoperatively. As time progresses, the unique advantages of multiple arterial revascularizations may gradually become apparent, and these advantages might not have been fully captured in the short-term follow-up. Third, in the present study the lack of differences observed would suggest that an appropriately powered definitive study based on short-term outcome between MAG and SAG would need to be prohibitively large. This is best illustrated by findings from large registries were the benefits increase with time but only become apparent after 10 years (27). This means that longer-term clinical endpoints are likely most appropriate for the design of any future RCT to compare grafting strategies in CABG.

Consistent with other studies (28, 29), renal impairment confers a significantly increased operative mortality and post-operative morbidity risk, including MACCEs, IABP utilization, and postoperative dialysis. This may be attributed to the impaired metabolic and regulatory functions in patients with chronic renal insufficiency, leading to reduced tolerance to surgery and anesthesia and an increased susceptibility to various complications (30, 31). Further, patients with CKD typically have more diseased and heavily calcified vessels, perhaps evidenced by the higher prevalence of vascular disease associated with lower pre-operative GFRs amongst our cohort. This too may account for the increased mortality. Such issues make CABG technically more difficult with poor target vessels, increasing the duration of the procedure, thereby increasing the mortality and complication rate. Moreover, a good LVEF can, to a certain extent, protect patients from the impacts of postoperative dialysis, MACCEs, and IABP application. This further underscores the importance of preoperative LVEF evaluation, as patients with well-preserved left ventricular function may be more suitable candidates for surgical treatment and exhibit a relatively lower risk of postoperative complications (32, 33). Therefore, for such patients, a comprehensive preoperative assessment and optimized treatment strategies for cardiac and renal function should be implemented to mitigate surgical risks.

Mechanical ventilation duration is correlated with the risk of postoperative dialysis and MACCEs. Prolonged mechanical ventilation may elevate the risk of complications such as pulmonary infections, thereby influencing the overall prognosis of patients (34, 35). Consequently, efforts should be made to minimize mechanical ventilation time postoperatively and promote the recovery of spontaneous breathing. The presence of left main coronary artery disease (LMCAD) serves as a predictive factor for re-thoracotomy, potentially due to the severity and complexity of LMCAD, which renders the surgical procedure more challenging and increases the likelihood of postoperative complications such as bleeding, necessitating re-thoracotomy (36, 37). For these patients, a detailed preoperative surgical plan should be formulated, and meticulous intraoperative manipulation should be exercised to reduce the risk of re-thoracotomy.

The clinical outcome superiority between on-pump CABG and off-pump CABG has been a persistent controversy. Some studies have found that off-pump CABG could reduce the incidence of postoperative adverse events in the early 21st century (38). Large-scale database studies demonstrated that off-pump CABG could lower the operative mortality rate in high-risk patients and improve long-term survival rates (39–41). However, a substantial number of clinical RCTs conducted in recent years have suggested that on-pump CABG offers better early- to mid-term prognosis compared to off-pump CABG (42, 43). In our study, on-pump status did not have a significant impact on in-hospital adverse events, except for the influence of potential confounders, which may be related to the advancements in surgical techniques, cardiopulmonary bypass technology, anesthesia techniques, and the level of intensive care medical services. In the future, large-scale RCTs, along with long-term follow-up data, will be required to further investigate the impact of off-pump and on-pump approaches on long-term outcomes.

Several limitations warrant consideration in this study. First, in terms of the single-center nature, our hospital primarily serves patients from a specific geographical region. The disease prevalence, risk factors, and even patients' treatment adherence in this area may differ significantly from those in other regions, making the patient population in our center not fully representative of the broader population. Moreover, the clinical practices and expertise within our medical team are unique to our institution. The diagnostic criteria, treatment protocols, and surgical techniques employed here may not be identical to those in other hospitals, which could lead to different treatment outcomes. Thus, for centers with significantly different patient populations or clinical environments, the generalization of our results may be limited. Second, as a retrospective study, we only rely on existing medical records for data collection. Some unmeasured factors, such as surgeon experience, operation time and completeness of revascularization, were not systematically recorded and accounted for in our retrospective data collection process. Although we employed a generalized mixed-effects multivariable logistic regression and sensitivity analysis to minimize confounding during data analysis, the non-randomized design inherently limits our ability to account for all potential confounders, which could introduce residual bias into the findings. Moreover, due to the objective limitations of data collection in this study, the number of events in some categories was relatively small. This increased the uncertainty in model parameter estimation and widening the confidence intervals. Future research will require more data to increase the number of events, thereby enhancing the precision of estimates and narrowing the confidence intervals. Third, most current large-scale RCT studies have already demonstrated the long-term benefits of MAG revascularization (16–19), but our study only focused on short-term outcomes without finding significant differences, we acknowledge the limited novelty of our research. However, this does not negate the potential long-term benefits of MAG revascularization. In fact, the results of our study do not conflict with those of large multi-center studies. Instead, we provide Supplementary Material on the effects of MAG revascularization from different time perspectives. Forth, our study lacks analysis of specific subgroups. In the future study, specialized comparison on the specific subgroups, such as patients with diabetes, those with reduced left ventricular ejection fraction, dialysis patients, and patients with complex anatomical structures, should be carried out to uncover potentially more valuable information regarding the two treatment approaches in these special groups.

In conclusion, our study did not find significant differences in short-term outcomes between MAG and SAG groups. However, caution should be exercised when applying these findings to other clinical environments and patient populations. Further multi-center, prospective RCTs are needed to validate and extend our results. Moreover, factors such as chronic renal insufficiency, poor LVEF, prolonged mechanical ventilation duration, and the presence of LMCAD significantly increased the risk of postoperative adverse outcomes. These data highlight the importance of pre-operative evaluation and optimization of LVEF and renal function, while further guiding clinicians in refining ventilation strategies and LMCAD management to minimize postoperative adverse events.

## Data Availability

The original contributions presented in the study are included in the article/Supplementary Material, further inquiries can be directed to the corresponding authors.
