# Supplementary material for: Comparison in trends and outcomes of multiple vs. single arterial coronary bypass graft surgery
Source: Front Cardiovasc Med. 2025 Nov 5;12:1661006. doi: 10.3389/fcvm.2025.1661006 (PMC12626920; doi:10.3389/fcvm.2025.1661006)
Supplement: Supplementary file 1 [file Datasheet1.pdf]

Comparison in trends and outcomes of multiple versus single arterial coronary bypass graft surgery

Qiuju Ding[#1], Han Li[#1], Xiaofeng Cheng[*1.2], Min Ge[*1], Qing Zhou[*1]

[1]Department of Cardio-thoracic Surgery, Nanjing Drum Tower Hospital, The Affiliated Hospital of Nanjing University Medical School, Nanjing, China

[2]Department of Cardio-Thoracic Surgery, Nanjing Drum Tower Hospital, Clinical College of Nanjing University of Chinese Medicine, Nanjing, China.

[#]These authors contributed equally.

***Corresponding author:**

Xiaofeng Cheng, Ph.D., M.D.

Department of Cardio-thoracic Surgery, Nanjing Drum Tower Hospital, The Affiliated Hospital of Nanjing University Medical School, Zhongshan Road 215, Nanjing 210008, China.

E-mail: chengxiaofeng_glyy@163.com

Min Ge, Ph.D., M.D.

Department of Cardio-thoracic Surgery, Nanjing Drum Tower Hospital, The Affiliated Hospital of Nanjing University Medical School, Zhongshan Road 321, Nanjing 210008, China.

Email: gemin2000@outlook.com

Qing Zhou, Ph.D., M.D.

Department of Cardio-thoracic Surgery, Nanjing Drum Tower Hospital, The Affiliated Hospital of Nanjing University Medical School, Zhongshan Road 321, Nanjing 210008, China.

Email: zhouqing_penn@163.com

Supplementary Table S1. Effect estimates were calculated using a generalized mixed-effects model to establish the effect of specific perioperative covariates upon in-hospital MACCE in patients receiving CABG.

Variable	OR	95% CI	P-value
(Intercept)	0.000	[0-0.01]	<0.001
MAG group	1.621	[0.563-4.665]	0.371
Male	1.865	[0.552-6.301]	0.316
Age	1.030	[0.98-1.083]	0.248
BMI	1.082	[0.939-1.246]	0.275
Smoking history	0.646	[0.202-2.062]	0.46
Drinking history	1.162	[0.308-4.381]	0.825
Neurological dysfunction	1.471	[0.525-4.116]	0.463
CKD	4.576	[1.492-14.033]	0.008
Hypertension	2.608	[0.688-9.881]	0.158
Diabetes	0.969	[0.373-2.515]	0.949
Hyperlipidemia	1.154	[0.418-3.184]	0.782
Valvular disease	1.971	[0.21-18.494]	0.552
Atrial fibrillation	0.831	[0.108-6.399]	0.859
Peripheral vascular disease	0.760	[0.217-2.665]	0.668
COPD	0.007	[0-891122250.091]	0.705
Pulmonary disease	0.000	[0-Inf]	0.996
NYHA IV	1.885	[0.369-9.643]	0.446
Poor LVEF (EF<30%)	13.785	[1.76-107.975]	0.012
Recent MI	1.375	[0.514-3.677]	0.526
Prior PCI	1.428	[0.475-4.292]	0.525
Left main coronary artery	1.700	[0.66-4.381]	0.272
Three vessel disease	1.160	[0.392-3.435]	0.788
Cardiopulmonary by pass	1.318	[0.473-3.673]	0.598
Tracheal intubation time	1.014	[1.001-1.026]	0.029
ICU stay	1.096	[0.982-1.222]	0.102

Abbreviation: BMI: body mass index; CI: Confidence Interval; CICU: cardiac surgical intensive care unit; CKD: Chronic kidney disease; COPD: chronic obstructive pulmonary disease; LVEF: left ventricular ejection fraction; IABP: intra-aortic balloon pump; MI: myocardial infarction; NYHA: New York Heart Association; OR: Odds Ratio; PCI: percutaneous coronary intervention.

Supplementary Table S2. Effect estimates were calculated using a generalized mixed-effects model to establish the effect of specific perioperative covariates upon IABP use in patients receiving CABG.

Variable	OR	95% CI	P-value
(Intercept)	0.000	[0-0.198]	0.018
MAG group	1.069	[0.215-5.305]	0.935
Male	0.722	[0.135-3.876]	0.704
Age	1.010	[0.946-1.077]	0.77
BMI	1.075	[0.877-1.317]	0.487
Smoking history	1.412	[0.291-6.85]	0.669
Drinking history	0.749	[0.113-4.972]	0.764
Neurological dysfunction	0.836	[0.165-4.227]	0.829
CKD	8.306	[1.721-40.084]	0.008
Hypertension	3.095	[0.492-19.464]	0.228
Diabetes	0.495	[0.125-1.97]	0.319
Hyperlipidemia	3.047	[0.746-12.446]	0.121
Valvular disease	0.000	[0-Inf]	0.998
Atrial fibrillation	3.239	[0.339-30.933]	0.307
Peripheral vascular disease	0.665	[0.124-3.568]	0.634
COPD	0.042	[0-217.079]	0.467
Pulmonary disease	0.000	[0-Inf]	0.997
NYHA IV	0.550	[0.043-7.093]	0.646
Poor LVEF (EF<30%)	50.032	[3.531-708.941]	0.004
Recent MI	2.282	[0.578-9.017]	0.239
Prior PCI	1.404	[0.302-6.526]	0.665
Left main coronary artery	2.776	[0.74-10.407]	0.13
Three vessel disease	0.490	[0.122-1.963]	0.314
Cardiopulmonary by pass	2.212	[0.572-8.559]	0.25
Tracheal intubation time	1.009	[0.996-1.022]	0.184
ICU stay	1.094	[0.97-1.234]	0.144

Abbreviation: BMI: body mass index; CI: Confidence Interval; CICU: cardiac surgical intensive care unit; CKD: Chronic kidney disease; COPD: chronic obstructive pulmonary disease; LVEF: left ventricular ejection fraction; IABP: intra-aortic balloon pump; MI: myocardial infarction; NYHA: New York Heart Association; OR: Odds Ratio; PCI: percutaneous coronary intervention.

Supplementary Table S3. Effect estimates were calculated using a generalized mixed-effects model to establish the effect of specific perioperative covariates upon postoperative dialysis in patients receiving CABG.

Variable	OR	95% CI	P-value
(Intercept)	0.000	[0-0.011]	<0.001
MAG group	0.576	[0.146-2.277]	0.431
Male	1.182	[0.34-4.111]	0.793
Age	1.021	[0.966-1.079]	0.466
BMI	1.132	[0.97-1.322]	0.116
Smoking history	1.282	[0.334-4.914]	0.718
Drinking history	1.045	[0.241-4.525]	0.953
Neurological dysfunction	2.302	[0.779-6.801]	0.132
CKD	16.957	[5.863-49.045]	<0.001
Hypertension	4.443	[0.669-29.52]	0.123
Diabetes	1.316	[0.453-3.824]	0.614
Hyperlipidemia	0.913	[0.295-2.824]	0.875
Valvular disease	1.972	[0.113-34.482]	0.642
Atrial fibrillation	0.000	[0-Inf]	0.997
Peripheral vascular disease	0.282	[0.047-1.683]	0.165
COPD	0.011	[0-7459408.37]	0.662
Pulmonary disease	0.815	[0.049-13.513]	0.886
NYHA IV	0.183	[0.011-3.037]	0.236
Poor LVEF (EF<30%)	57.398	[3.755-877.381]	0.004
Recent MI	1.110	[0.352-3.498]	0.859
Prior PCI	1.266	[0.362-4.431]	0.712
Left main coronary artery	1.360	[0.446-4.148]	0.589
Three vessel disease	0.561	[0.196-1.606]	0.281
Cardiopulmonary by pass	0.954	[0.291-3.123]	0.938
Tracheal intubation time	1.015	[1.001-1.029]	0.037
ICU stay	1.026	[0.906-1.163]	0.683

Abbreviation: BMI: body mass index; CI: Confidence Interval; CICU: cardiac surgical intensive care unit; CKD: Chronic kidney disease; COPD: chronic obstructive pulmonary disease; LVEF: left ventricular ejection fraction; IABP: intra-aortic balloon pump; MI: myocardial infarction; NYHA: New York Heart Association; OR: Odds Ratio; PCI: percutaneous coronary intervention.

Supplementary Table S4. Effect estimates were calculated using a generalized mixed-effects model to establish the effect of specific perioperative covariates upon re-thoracotomy for bleeding in patients receiving CABG.

Variable	OR	95% CI	P-value
(Intercept)	0.018	[0-39.012]	0.304
MAG group	0.422	[0.069-2.561]	0.348
Male	2.543	[0.248-26.107]	0.432
Age	0.965	[0.895-1.04]	0.349
BMI	0.986	[0.81-1.201]	0.891
Smoking history	1.194	[0.283-5.031]	0.809
Drinking history	1.949	[0.431-8.807]	0.386
Neurological dysfunction	2.630	[0.573-12.064]	0.213
CKD	1.472	[0.088-24.633]	0.788
Hypertension	0.662	[0.154-2.843]	0.579
Diabetes	0.372	[0.091-1.523]	0.169
Hyperlipidemia	0.161	[0.018-1.452]	0.104
Valvular disease	0.000	[0-Inf]	0.999
Atrial fibrillation	0.000	[0-Inf]	0.999
Peripheral vascular disease	0.925	[0.16-5.353]	0.93
COPD	2.262	[0.195-26.258]	0.514
Pulmonary disease	0.000	[0-Inf]	0.999
NYHA IV	3.123	[0.209-46.657]	0.409
Poor LVEF (EF<30%)	0.000	[0-Inf]	0.999
Recent MI	2.181	[0.556-8.566]	0.264
Prior PCI	2.716	[0.633-11.664]	0.179
Left main coronary artery	4.956	[1.306-18.801]	0.019
Three vessel disease	2.782	[0.508-15.222]	0.238
Cardiopulmonary by pass	0.251	[0.027-2.332]	0.224
Tracheal intubation time	1.002	[0.983-1.021]	0.844
ICU stay	1.050	[0.837-1.318]	0.671

Abbreviation: BMI: body mass index; CI: Confidence Interval; CICU: cardiac surgical intensive care unit; CKD: Chronic kidney disease; COPD: chronic obstructive pulmonary disease; LVEF: left ventricular ejection fraction; IABP: intra-aortic balloon pump; MI: myocardial infarction; NYHA: New York Heart Association; OR: Odds Ratio; PCI: percutaneous coronary intervention.

Supplementary Table S5. Effect estimates were calculated using a generalized mixed-effects model to establish the effect of specific perioperative covariates upon sterile wound dehiscence in patients receiving CABG.

Variable	OR	95% CI	P-value
(Intercept)	0.004	[0-0.265]	0.009
MAG group	1.313	[0.601-2.872]	0.495
Male	0.489	[0.221-1.085]	0.079
Age	1.019	[0.979-1.06]	0.362
BMI	1.046	[0.943-1.161]	0.393
Smoking history	1.473	[0.635-3.417]	0.367
Drinking history	1.038	[0.383-2.816]	0.941
Neurological dysfunction	1.499	[0.696-3.228]	0.301
CKD	1.309	[0.347-4.943]	0.691
Hypertension	0.866	[0.408-1.841]	0.709
Diabetes	1.783	[0.894-3.555]	0.1
Hyperlipidemia	0.995	[0.476-2.077]	0.989
Valvular disease	1.047	[0.121-9.021]	0.967
Atrial fibrillation	0.549	[0.064-4.731]	0.586
Peripheral vascular disease	0.996	[0.414-2.398]	0.994
COPD	1.018	[0.144-7.17]	0.986
Pulmonary disease	1.263	[0.269-5.93]	0.768
NYHA IV	2.077	[0.53-8.137]	0.294
Poor LVEF (EF<30%)	0.000	[0-Inf]	0.996
Recent MI	1.088	[0.475-2.493]	0.843
Prior PCI	1.099	[0.479-2.522]	0.824
Left main coronary artery	1.280	[0.627-2.613]	0.498
Three vessel disease	0.829	[0.397-1.729]	0.617
Cardiopulmonary by pass	0.670	[0.27-1.661]	0.387
Tracheal intubation time	1.008	[0.997-1.019]	0.151
ICU stay	0.953	[0.827-1.098]	0.504

Abbreviation: BMI: body mass index; CI: Confidence Interval; CICU: cardiac surgical intensive care unit; CKD: Chronic kidney disease; COPD: chronic obstructive pulmonary disease; LVEF: left ventricular ejection fraction; IABP: intra-aortic balloon pump; MI: myocardial infarction; NYHA: New York Heart Association; OR: Odds Ratio; PCI: percutaneous coronary intervention.

Supplementary Table S6. Sensitivity analysis assesses the contribution of each fixed effect variable to in-hospital MACCE in patients receiving CABG.

Variable	df	AIC	LRT	P-value
<none>		240.88		
MAG group	1	239.70	0.820	0.365
Male	1	240.01	1.132	0.287
Age	1	240.36	1.477	0.224
BMI	1	240.15	1.273	0.259
Smoking history	1	239.45	0.572	0.449
Drinking history	1	238.93	0.050	0.822
Neurological dysfunction	1	239.41	0.534	0.465
CKD	1	245.29	6.407	0.011
Hypertension	1	241.33	2.451	0.117
Diabetes	1	238.88	0.003	0.955
Hyperlipidemia	1	238.96	0.078	0.78
Valvular disease	1	239.20	0.322	0.571
Atrial fibrillation	1	238.91	0.030	0.863
Peripheral vascular disease	1	239.08	0.198	0.656
COPD	1	239.73	0.854	0.355
Pulmonary disease	1	243.49	4.615	0.032
NYHA IV	1	239.43	0.554	0.457
Poor LVEF (EF<30%)	1	244.41	5.532	0.019
Recent MI	1	239.28	0.405	0.524
Prior PCI	1	239.28	0.402	0.526
Left main coronary artery	1	240.09	1.212	0.271
Three vessel disease	1	238.96	0.080	0.777
Cardiopulmonary by pass	1	239.16	0.277	0.598
Tracheal intubation time	1	244.19	5.314	0.021
ICU stay	1	241.65	2.770	0.096

Abbreviation: AIC, Akaike Information Criterion; df, degrees of freedom; LRT, Likelihood Ratio Test Statistic. Lower AIC indicates better model fit. P-value < 0.05 indicates significant contribution.

Supplementary Table S7. Sensitivity analysis assesses the contribution of each fixed effect variable to IABP use in patients receiving CABG.

Variable	df	AIC	LRT	P-value
<none>		169.74		
MAG group	1	167.76	0.013	0.908
Male	1	167.89	0.147	0.701
Age	1	167.89	0.146	0.703
BMI	1	168.41	0.663	0.416
Smoking history	1	167.96	0.218	0.641
Drinking history	1	167.85	0.104	0.747
Neurological dysfunction	1	167.79	0.051	0.821
CKD	1	174.46	6.718	0.01
Hypertension	1	169.62	1.879	0.17
Diabetes	1	168.91	1.171	0.279
Hyperlipidemia	1	170.40	2.657	0.103
Valvular disease	1	168.47	0.725	0.395
Atrial fibrillation	1	168.66	0.918	0.338
Peripheral vascular disease	1	168.01	0.263	0.608
COPD	1	168.90	1.153	0.283
Pulmonary disease	1	171.06	3.315	0.069
NYHA IV	1	167.99	0.242	0.623
Poor LVEF (EF<30%)	1	176.28	8.540	0.003
Recent MI	1	169.19	1.447	0.229
Prior PCI	1	167.94	0.194	0.66
Left main coronary artery	1	170.27	2.523	0.112
Three vessel disease	1	168.82	1.073	0.3
Cardiopulmonary by pass	1	169.09	1.346	0.246
Tracheal intubation time	1	169.78	2.041	0.153
ICU stay	1	170.01	2.266	0.132

Abbreviation: AIC, Akaike Information Criterion; df, degrees of freedom; LRT, Likelihood Ratio Test Statistic. Lower AIC indicates better model fit. P-value < 0.05 indicates significant contribution.

Supplementary Table S8. Sensitivity analysis assesses the contribution of each fixed effect variable to postoperative dialysis in patients receiving CABG.

Variable	df	AIC	LRT	P-value
<none>		218.47		
MAG group	1	217.15	0.676	0.411
Male	1	216.56	0.086	0.769
Age	1	217.16	0.684	0.408
BMI	1	219.28	2.809	0.094
Smoking history	1	216.62	0.148	0.7
Drinking history	1	216.47	0.004	0.951
Neurological dysfunction	1	218.78	2.306	0.129
CKD	1	242.51	26.036	<0.001
Hypertension	1	219.85	3.381	0.066
Diabetes	1	216.75	0.284	0.594
Hyperlipidemia	1	216.50	0.027	0.87
Valvular disease	1	216.67	0.195	0.659
Atrial fibrillation	1	217.87	1.399	0.237
Peripheral vascular disease	1	218.99	2.524	0.112
COPD	1	217.52	1.050	0.305
Pulmonary disease	1	216.49	0.021	0.885
NYHA IV	1	218.18	1.714	0.191
Poor LVEF (EF<30%)	1	225.14	8.669	0.003
Recent MI	1	216.50	0.029	0.866
Prior PCI	1	216.62	0.145	0.703
Left main coronary artery	1	216.77	0.300	0.584
Three vessel disease	1	217.62	1.154	0.283
Cardiopulmonary by pass	1	216.48	0.006	0.94
Tracheal intubation time	1	221.33	4.856	0.028
ICU stay	1	216.64	0.164	0.685

Abbreviation: AIC, Akaike Information Criterion; df, degrees of freedom; LRT, Likelihood Ratio Test Statistic. Lower AIC indicates better model fit. P-value < 0.05 indicates significant contribution.

Supplementary Table S9. Sensitivity analysis assesses the contribution of each fixed effect variable to re-thoracotomy for bleeding in patients receiving CABG.

Variable	df	AIC	LRT	P-value
<none>		156.57		
MAG group	1	155.59	1.017	0.313
Male	1	155.33	0.761	0.383
Age	1	155.44	0.869	0.351
BMI	1	154.59	0.016	0.9
Smoking history	1	154.63	0.061	0.805
Drinking history	1	155.31	0.744	0.388
Neurological dysfunction	1	156.02	1.450	0.228
CKD	1	154.64	0.071	0.791
Hypertension	1	154.88	0.312	0.576
Diabetes	1	156.64	2.067	0.151
Hyperlipidemia	1	158.76	4.186	0.041
Valvular disease	1	154.92	0.347	0.556
Atrial fibrillation	1	155.53	0.957	0.328
Peripheral vascular disease	1	154.58	0.008	0.928
COPD	1	154.96	0.386	0.535
Pulmonary disease	1	156.77	2.195	0.138
NYHA IV	1	155.17	0.595	0.44
Poor LVEF (EF<30%)	1	155.12	0.552	0.458
Recent MI	1	155.79	1.223	0.269
Prior PCI	1	156.30	1.727	0.189
Left main coronary artery	1	160.60	6.026	0.014
Three vessel disease	1	156.26	1.690	0.194
Cardiopulmonary by pass	1	156.67	2.102	0.147
Tracheal intubation time	1	154.61	0.037	0.848
ICU stay	1	154.74	0.174	0.676

Abbreviation: AIC, Akaike Information Criterion; df, degrees of freedom; LRT, Likelihood Ratio Test Statistic. Lower AIC indicates better model fit. P-value < 0.05 indicates significant contribution.

Supplementary Table S10. Sensitivity analysis assesses the contribution of each fixed effect variable to sterile wound dehiscence in patients receiving CABG.

Variable	df	AIC	LRT	P-value
<none>		386.29		
MAG group	1	384.78	0.483	0.487
Male	1	387.44	3.145	0.076
Age	1	385.24	0.946	0.331
BMI	1	385.10	0.802	0.371
Smoking history	1	385.14	0.845	0.358
Drinking history	1	384.30	0.005	0.941
Neurological dysfunction	1	385.35	1.057	0.304
CKD	1	384.45	0.154	0.695
Hypertension	1	384.44	0.142	0.706
Diabetes	1	387.16	2.867	0.09
Hyperlipidemia	1	384.29	0.000	1
Valvular disease	1	384.30	0.002	0.966
Atrial fibrillation	1	384.66	0.366	0.545
Peripheral vascular disease	1	384.29	0.000	1
COPD	1	384.29	0.000	0.987
Pulmonary disease	1	384.38	0.085	0.771
NYHA IV	1	385.29	0.997	0.318
Poor LVEF (EF<30%)	1	385.44	1.145	0.285
Recent MI	1	384.34	0.041	0.839
Prior PCI	1	384.35	0.051	0.822
Left main coronary artery	1	384.76	0.466	0.495
Three vessel disease	1	384.54	0.249	0.618
Cardiopulmonary by pass	1	385.12	0.828	0.363
Tracheal intubation time	1	386.94	2.641	0.104
ICU stay	1	384.82	0.524	0.469

Abbreviation: AIC, Akaike Information Criterion; df, degrees of freedom; LRT, Likelihood Ratio Test Statistic. Lower AIC indicates better model fit. P-value < 0.05 indicates significant contribution.
